# Zenana Medical Mission

**Published:** 1890-09-13

**Authors:** G. de G. Griffith

**Affiliations:** Hon. Sec. (M.A.R.) Zenana Medical College, 58. St. George's Road, S.W.


### Editor's Letter-Box.

[Our Correspondents are reminded that prolixity is a great bar to publication, and that brevity of style and conciseness of statement greatly facilitate early insertion.]

### ZENANA MEDICAL MISSION.

Sir—Will you kindly do us the favour of allowing the accompanying letter to find a space in your interesting and valuable paper? It will be a help to us if you will do so.—Very faithfully yours,

G. de G. Griffith, Hon. Sec. (M.A.R.)
Zenana Medical College, 58, St. George's Road, S.W.

Deritsyah, August 7th, 1890.

I have been kept very busy, as we have had a good deal of influenza and tonsilitis during the winter. I prescribed for over 300 patients, and visited over 100 in their own homes. I have had abundant success, and can never be thankful enough that I was led to study medicine. I should like to have another year at the college when I go home. It has been a great help to me in my work; indeed, all the pupils in my school have come because I attended some member of their families when ill. Even the local Governor called on me for sore throat, and also for his family. I had so run down, I had to come here for a change; it is a very pleasant village, about twenty hours from Damascus, and much drier than Swediah. Of course it soon leaked out who I was, and I have been nearly as much beset with patients as I was at home. Fortunately I brought no medicine with me, and when they hear that, their number diminishes somewhat. Hoping soon to hear from you again, believe me, yours very sincerely, Meta Cunningham.

